# A Novel *ATRIP* Mutation Detected in an Iranian Family with Familial Clustering of Breast Cancer: A Case Report

**DOI:** 10.3390/curroncol32120711

**Published:** 2025-12-17

**Authors:** Neda Zamani, Mehar Chahal, Iman Salahshourifar, Reiyhane Talebian, Mohammad R. Akbari

**Affiliations:** 1 Women’s College Research Institute, Women’s College Hospital, University of Toronto, Toronto, ON M5S 1B2, Canada; neda.zamani@wchospital.ca (N.Z.); mehar.chahal@wchospital.ca (M.C.); 2 Institute of Medical Science, Faculty of Medicine, University of Toronto, Toronto, ON M5S 3H2, Canada; 3 Department of Biology, Science and Research Branch, Islamic Azad University, Tehran 1477893855, Iran; isalahshouri@iau.ac.ir; 4 Farda Pathobiology & Genetic Laboratory, Tehran 1953774641, Iran; re.talebian@iau.ac.ir; 5 Dalla Lana School of Public Health, University of Toronto, Toronto, ON M5T 3M7, Canada

**Keywords:** *ATRIP* mutation, genetic testing, hereditary breast cancer, family pedigree, case report

## Abstract

**Purpose:** ATRIP (ATR-interacting protein) is a critical partner of ATR (ataxia telangiectasia and Rad3-related). The ATR-ATRIP heterodimer plays an essential role in initiating homologous recombination repair (HRR) during replication stress and inducing double-stranded DNA breaks following unresolved stalled replication forks. Our team recently identified *ATRIP* as a novel breast cancer susceptibility gene candidate through whole-exome sequencing (WES) of familial breast cancer patients and healthy controls from the Polish founder population, with subsequent validation in both Polish and British cohorts. In the present study, we report for the first time the detection of a novel deleterious mutation in *ATRIP* among several members of an Iranian family with clustering of breast cancer who were negative for mutations in the already known breast cancer risk genes. **Methods:** Six family members underwent germline DNA testing by WES, following initial negative results from multigene panel testing. Candidate variants were confirmed by Sanger sequencing and assessed according to ACMG guidelines. **Results:** We detected a novel *ATRIP* frameshift mutation (NM_130384.3:c.1033delC) in four of six family members that were tested, including two individuals affected with breast cancer. No pathogenic variants were found in other known cancer susceptibility genes. **Conclusions:** This is the first report of a deleterious *ATRIP* mutation in an Iranian family with familial breast cancer, suggesting a potential role of *ATRIP* in hereditary breast cancer. Further studies are required to confirm the role of ATRIP in breast cancer susceptibility, refine risk assessment, and evaluate potential personalized therapeutic strategies. In the interim, genetic counseling for ATRIP mutation carriers should proceed with caution, given current limitations in clinical interpretation.

## Simple Summary

Breast cancer can run in families when harmful mutations in certain genes are passed from one generation to the next. Many genes linked to inherited breast cancer are already known, but they do not explain all families with clustering of the disease. Our research team recently identified *ATRIP* as a novel gene that may increase the risk of developing breast cancer. In this report, we describe an Iranian family with several close relatives diagnosed with breast cancer, yet no deleterious mutations were found in the commonly tested breast cancer genes. Using advanced genetic testing, we discovered a harmful *ATRIP* mutation shared by multiple family members, including two who had breast cancer. This finding supports the growing evidence that *ATRIP* may play an important role in hereditary breast cancer. More research is needed to understand the risks and to guide screening and prevention for families harboring such mutations.

## 1. Introduction

Breast cancer remains the most commonly diagnosed malignancy among women worldwide and is a major contributor to cancer-related deaths [1]. The development of breast cancer is influenced by a combination of various modifiable and non-modifiable risk factors, among which genetic factors have a significant role in susceptibility to the disease. Approximately 5–10% of all breast cancer cases are attributed to inherited predisposition. Among hereditary cases, deleterious mutations in the well-established genes *BRCA1* and *BRCA2* account for roughly 20–25% of known familial cases [2,3]. Beyond *BRCA1/2*, several other genes—including *ATM*, *BARD1*, *CHEK2*, *PALB2*, *RAD51C*, *RAD51D*, and *TP53*—have been recognized as moderate- to high-penetrance genes associated with increased breast cancer susceptibility [4,5].

Despite these findings, a significant proportion of families with high breast cancer clustering remains genetically unexplained, indicating that additional susceptibility genes are yet to be identified. Advances in next-generation sequencing technologies have sped up the search for novel hereditary breast cancer genes in recent years. However, the clinical interpretation of these emerging candidate genes is still evolving, and questions about their penetrance and pathogenic mechanisms continue to be investigated [6].

In some families, the pattern of cancer strongly indicates an inherited etiology, yet routine multigene panel testing yields negative or inconclusive results. In such scenarios, further evaluation at specialized hereditary cancer genetics centers can be essential. These centers often have access to research-based sequencing platforms, such as whole-exome or whole-genome sequencing, that allow the detection of rare or newly emerging susceptibility gene candidates not yet incorporated into routine clinical panels. When clinical suspicion remains high, this deeper level of evaluation can help reduce false-negative results and ensure more comprehensive risk assessment and counseling.

Our research team has recently discovered *ATRIP* as a novel breast cancer susceptibility gene candidate by performing whole-exome sequencing (WES) on a cohort of highly familial breast cancer patients with unknown genetic predisposition and a control group of unaffected women from the founder population of Poland. At the validation, a rare recurrent mutation in *ATRIP*, c.1152_1155del, was observed in 42 of 16,085 Polish breast cancer patients and in 11 of 9285 controls (OR = 2.14, 95% CI = 1.13–4.28, *p* = 0.02) [7]. We further validated the association of *ATRIP* deleterious mutations with an increased risk of breast cancer using WES data of 15,643 Caucasian breast cancer patients and 157,943 ethnically matched cancer-free individuals from the UK Biobank (UKB) cohort study. LoF variants in *ATRIP* were observed in 13 cases and 40 healthy individuals within this cohort (age-adjusted OR = 3.28, 95% CI = 1.76–6.14, *p* < 0.001) [7].

ATRIP plays a central role in the ATR-mediated DNA damage response, a pathway essential for maintaining genomic stability, especially under replication stress. Disruption of this pathway may contribute to tumorigenesis [8], yet the extent to which *ATRIP* LoF variants contribute to hereditary breast cancer is still being elucidated. Evidence remains limited, and additional reports across diverse populations are needed to clarify the gene’s clinical significance.

The current study adds to this emerging body of literature by reporting, for the first time in an Iranian family, a truncating *ATRIP* variant identified in multiple members with a strong history of breast cancer who had previously tested negative for pathogenic variants in established breast cancer genes assessed through multigene panel testing. By documenting this variant in an Iranian family, we provide evidence that *ATRIP*-associated breast cancer susceptibility is not confined to European populations previously studied and may have broader global relevance, highlighting the importance of evaluating such novel risk candidate genes across diverse populations.

## 2. Case Description

**Figure 1:**
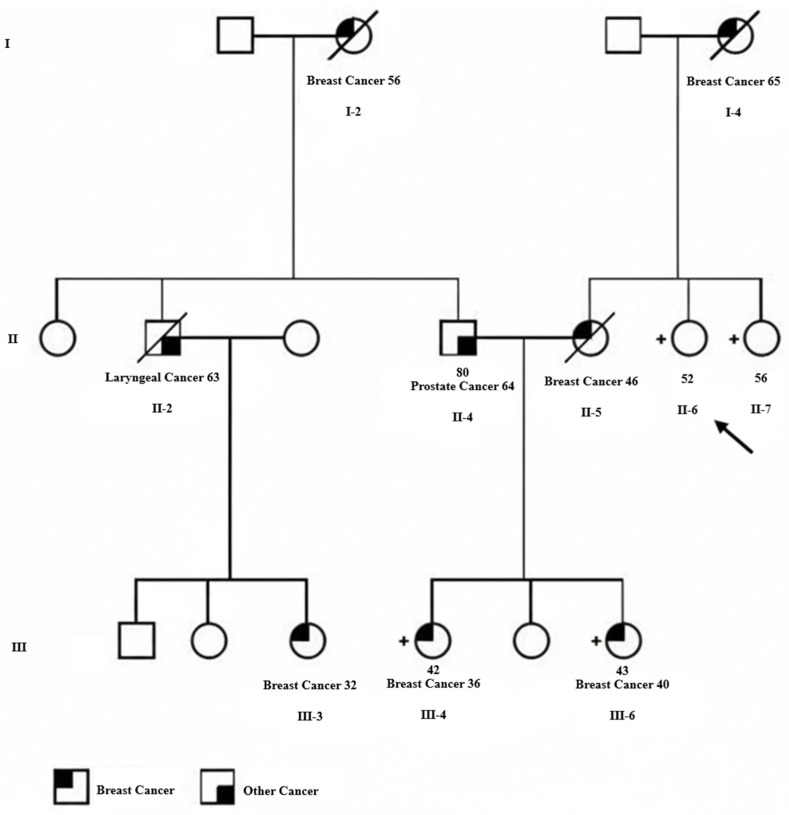
Pedigree for an Iranian family with clustering of breast cancer and carriers of the germline *ATRIP* c.1033delC mutation. The proband is shown with an arrow. The type of cancer and age at the time of diagnosis are indicated for individuals with cancer diagnosis. Square represents male and circle represents female. Deceased individuals are indicated by a diagonal line through the symbol. Carriers of the *ATRIP* c.1033delC mutation are indicated by a + sign next to the symbol.

**Figure 2:**
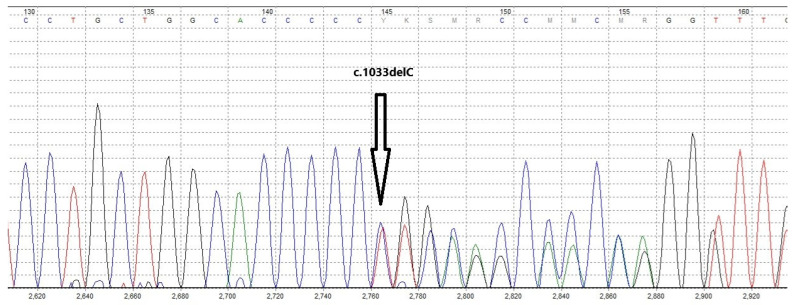
Sanger sequencing chromatogram of the proband’s genomic DNA confirming the presence of the germline *ATRIP* c.1033delC mutation. Different colored curves represent the four nucleotide bases in the chromatogram (A = green, C = blue, G = black, and T = red).

The proband (Figure 1, II-6) was a 52-year-old cancer-free Iranian woman who was referred to Farda Pathobiology & Genetic Laboratory (Tehran, Iran) due to concern regarding multiple first- and second-degree family members affected with breast cancer. She wanted to be screened for mutations in breast cancer susceptibility genes and be assessed for her risk of developing breast cancer. The proband’s mother (Figure 1, I-4) was diagnosed with grade III invasive ductal carcinoma of the left breast at the age of 65 years old, with the largest tumor dimension of 5 cm and extensive tumor necrosis. Immunohistochemical (IHC) staining revealed expression of the estrogen receptor (ER+) and lack of expression of the progesterone receptor (PR-). The patient died of metastatic complications. One of the proband’s sisters (Figure 1, II-5) was diagnosed with grade I invasive ductal carcinoma of the right breast at the age of 46 years old and died of cancer-related complications. Two of the three daughters of this patient (nieces of the proband, Figure 1, III-4 and III-6), whose details are unknown, developed invasive ductal carcinoma when they were 36 and 40 years old, respectively, and both were alive at the time of this study. The father of these two breast cancer-affected sisters (husband of the proband’s sister, Figure 1, II-4) also developed prostate cancer at the age of 64 years old, and his mother (Figure 1, I-2), who was not alive at the time of this study, was affected with breast cancer when she was 56 years old. Moreover, the paternal cousin of the proband’s affected nieces (Figure 1, III-3) was diagnosed with breast cancer at the age of 32 years old.

To unveil the underlying genetic predisposition, the proband was screened for mutations in a panel of 45 cancer-related genes, including the already known breast cancer susceptibility genes, in the Farda Pathobiology & Genetic Laboratory. The gene panel sequencing did not reveal any putatively deleterious mutations in the 45 cancer-related genes that were tested. Given the strong clinical suspicion of an inherited predisposition in this family despite negative multigene panel testing, the referring laboratory in Tehran sought additional evaluation from our Molecular Genetics Research Laboratory at Women’s College Hospital (Toronto, ON, Canada). Our group has expertise in investigating novel hereditary breast cancer gene candidates and in applying research-level sequencing approaches, including comprehensive whole-exome analysis, to unresolved familial cancer cases. Therefore, blood or saliva samples from six family members (Figure 1, II-4, II-6, II-7, III-3, III-4, and III-6) were sent to our laboratory for advanced genomic assessment, and WES was subsequently performed to further investigate the hereditary component of breast cancer in this family using NGS technology.

Briefly, germline DNA was isolated using standard methods from either peripheral blood leukocytes or saliva samples. The Agilent SureSelect human exome kit XT (V6) was used for library preparation and to capture target sequence regions. The SureSelect kit used has a target size of 62 Mb, and targets coding regions from RefSeq, CCDS and GENCODE known genes. The captured regions for each sample were barcoded and used for paired-end sequencing for 150 cycles on the Illumina NextSeq 500. The Sentieon software 202308.02 (Sentieon Inc., San Jose, CA, USA), which includes an optimized implementation of the Burrows-Wheeler Aligner (BWA) and Genome Analysis Toolkit (GATK), was used for secondary analysis of the sequencing data. The sequence reads were aligned to the human genome’s reference build hg19 (UCSC Genome Browser, February 2009 build). Regions with at least 20× depth of coverage were used for calling variants, and a different nucleotide from the reference sequence seen in at least 20% of the reads aligned to a given position was called as a variant. The SNP & Variation Suite (GoldenHelix Inc., Bozeman, MT, USA) was used for annotating called variants. We focused on LoF variants (frameshift insertions/deletions, stop codon gain, essential splicing site, and start codon loss variants) with a minor allele frequency (MAF) of ≤1% reported in gnomAD and 1000 Genomes Project databases. Identified LoF variants were assessed using the American College of Medical Genetics and Genomics/Association for Molecular Pathology (ACMG/AMP) guidelines for variant classification. Finally, variants classified as pathogenic/likely pathogenic were confirmed by Sanger sequencing.

Considering the very low mutation frequency of *ATRIP*, our newly discovered breast cancer susceptibility gene candidate, we did not expect to find an *ATRIP* mutation in this family and performed WES in search of other new candidate genes. But, surprisingly, we identified a deleterious mutation in this gene, a base pair deletion at position 1033 in the *ATRIP* coding sequence (NM_130384.3:c.1033delC), among four of the six tested family members. This mutation is classified as likely pathogenic according to the ACMG/AMP guidelines. Interestingly, all six exome-sequenced family members were negative for mutations in the already known breast or any other cancer susceptibility genes. The four *ATRIP* mutation carriers included the cancer-free proband (Figure 1, II-6), the proband’s unaffected sister (Figure 1, II-7), and the two breast cancer-affected nieces of the proband (Figure 1, III-4 & III-6). As the father of these two affected sisters (Figure 1, II-4) was not an *ATRIP* mutation carrier, we concluded that their mother (proband’s sister, Figure 1, II-5), who had died of breast cancer before this study, was the obligatory carrier of the *ATRIP* c.1033delC mutation. The *ATRIP* mutations identified by Illumina sequencing among the four family members were confirmed by Sanger sequencing (Figure 2). Unfortunately, tumor samples of the proband’s two breast cancer-affected nieces that harbored the *ATRIP* c.1033delC mutation were not available to perform additional studies, including loss of heterozygosity (LOH) analysis.

## 3. Discussion

In this case report, we describe, for the first time in an Iranian family, a truncating *ATRIP* variant detected in multiple members with strong clustering of breast cancer and no pathogenic variants in established hereditary breast cancer susceptibility genes. Although *ATRIP* pathogenic variants are very rare in the general population, our findings add to a growing body of evidence suggesting that deleterious variants in this gene may meaningfully contribute to inherited breast cancer predisposition.

Recently, our team introduced *ATRIP* as a novel breast cancer susceptibility gene associated with a higher risk of developing breast cancer with an OR of over 3 [8]. In addition to association studies, our team performed immunohistochemistry and functional analyses to compile more evidence regarding the pathogenicity of the *ATRIP* c.1152_1155del variant identified among the Polish population. These studies revealed that the *ATRIP* c.1152_1155del variant allele was weakly expressed compared to the wild-type allele, and the truncated protein failed to perform its normal function in preventing replicative stress [7]. Moreover, LOH analysis on the tumor DNA of ten available formalin-fixed paraffin-embedded (FFPE) samples of the *ATRIP* c.1152_1155del mutation carriers from Poland confirmed loss of the wild-type allele in four of ten tumor samples with the germline *ATRIP* c.1152_1155del variant. Also, seven of the ten *ATRIP*-mutated breast tumors, including those with confirmed LOH, had genomic homologous recombination repair deficiency (HRD), as defined by having an HRD score ≥ 42 [7].

Interestingly, after publishing our results and introducing *ATIRP* as a novel breast cancer susceptibility gene candidate, a meta-analysis across two other large WES datasets replicated the positive association of *ATRIP* deleterious mutations with the risk of developing breast cancer [9]. The datasets in this meta-analysis comprised the Breast Cancer Risk after Diagnostic Gene Sequencing (BRIDGES) dataset, including samples from eight studies in the Breast Cancer Association Consortium (BCAC) and the Personalized Risk Assessment for Prevention and Early Detection of Breast Cancer: Integration and Implementation (PERSPECTIVE) dataset, including three BCAC studies. Protein-truncating variants in *ATRIP* were observed among 13 of 8410 breast cancer cases and 3 of 8238 controls in these two datasets (OR = 3.69, 95% CI = 1.43–9.52, *p* = 0.007) [9].

Further support for the involvement of *ATRIP* in hereditary breast cancer predisposition has emerged from a large exome-wide gene burden meta-analysis published in 2025, which examined more than 74,000 breast cancer patients and 748,000 controls across multiple international cohorts, including the Million Women Study, UK Biobank, BCAC, All of Us, MGB, and FinnGen [10]. In this comprehensive analysis, *ATRIP* reached exome-wide significance for the first time, with protein-truncating variants conferring a statistically significant association with increased breast cancer risk, comparable in magnitude to other moderate-penetrance genes such as *BARD1*. Although *ATRIP* variants were rare, the aggregated burden across these large datasets consistently showed an elevated risk signal, reinforcing *ATRIP* as a genuine susceptibility gene rather than a population-specific or founder-limited observation [10]. The results of this large-scale analysis are consistent with our previous findings and further support the clinical relevance of *ATRIP* LoF variants, such as the c.1033delC mutation identified in our Iranian family. In total, although *ATRIP* LoF variants seem to be relatively rare, with a frequency of approximately 1 in every 1000–3000 of the general population, the significantly increased breast cancer risk associated with these variants suggests that *ATRIP* can be regarded as a moderately to highly penetrant gene in the context of hereditary breast cancer.

The identification of a deleterious *ATRIP* variant in the studied Iranian family supports the generalizability of previous findings beyond European populations. Importantly, all exome-sequenced family members were negative for pathogenic variants in the already known breast cancer risk genes, underscoring the potential contribution of *ATRIP*’s mutation to the familial clustering of the disease in this family. Although breast tumor tissue from the affected carriers was not available for LOH or HRD assessment, prior studies suggest that impaired ATRIP function may disrupt ATR-mediated checkpoint and the Fanconi anemia repair pathway, both of which are closely linked to HRR [11,12]. ATRIP deficiency compromises DNA replication fidelity, leading to chromosomal instability, impaired cell-cycle control, and reduced cell viability [8]. These mechanistic insights offer a biologically coherent explanation for how ATRIP loss may promote carcinogenesis.

This report also raises important clinical implications. As *ATRIP* is not routinely included in standard hereditary cancer genetic testing panels, families like the one presented here may remain genetically unexplained despite exhaustive multigene panel testing. Incorporation of *ATRIP* into such testing pipelines, particularly with the emergence of additional supportive evidence, may improve diagnostic yield for patients with hereditary breast cancer [13]. Furthermore, improved understanding of *ATRIP*-mutated tumor biology may ultimately help inform therapeutic decision-making.

Clinicians may occasionally encounter families in whom the pattern of cancer strongly suggests hereditary predisposition, yet multigene panel testing returns negative or inconclusive findings. In such situations, referral to specialized hereditary cancer genetics research centers should be considered, particularly those with access to research-based sequencing, broader variant curation resources, or gene-discovery programs. These centers can provide deeper genomic evaluation—such as whole-exome or whole-genome sequencing—and may identify rare or newly emerging susceptibility genes not yet included in routine clinical panels. Incorporating this additional layer of assessment is especially important when the clinical phenotype remains highly suggestive of an inherited etiology, as it helps reduce the likelihood of false-negative results and ensures that families receive more comprehensive risk assessment and counseling.

*ATRIP*, located on chromosome 3p21, plays a key role in the cellular response to DNA damage. When DNA lesions or stalled replication forks arise, single-stranded DNA (ssDNA) becomes rapidly coated by replication protein A (RPA), forming a protective complex on the exposed strand [14]. This RPA-ssDNA structure serves as a platform to recruit ATRIP, which in turn enables the ATR kinase to localize to the damaged region through its interaction with ATRIP. Assembly of the ATR–ATRIP complex at these sites facilitates the activation of CHEK1, a central effector of the DNA damage checkpoint, leading to temporary cell-cycle arrest and orchestration of the replication-stress response [15]. Germline pathogenic variants in *ATRIP* and *ATR* are known to cause Seckel syndrome, a rare disorder characterized by growth impairment and neurodevelopmental abnormalities, and *ATR* variants have also been linked to several cancer types, including oropharyngeal, skin, cervical, and breast cancers [16,17].

Experimental depletion of ATRIP in human cell lines using small interfering RNA results in destabilization of ATR and impaired activation of ATR-dependent checkpoint signaling following DNA damage. These findings support the concept that the RPA-ssDNA-ATRIP interaction is essential for efficient recruitment of ATR and initiation of downstream signaling events [18,19]. Although we previously showed that most *ATRIP*-mutated tumors have HRD, the direct association between loss of ATRIP function and resultant HRD in *ATRIP*-mutated tumor cells is indeed an area warranting further investigation. However, there are studies that provide indirect support for this association. For example, conditional ATRIP knockout models lacking ATR-interaction domains show markedly reduced monoubiquitination of FANCD2 and FANCI, as well as loss of FANCI phosphorylation—biochemical changes that imply potential disruption of the Fanconi anemia DNA repair pathway by loss of ATRIP function [11,12]. It has been shown that defects in this pathway may lead to compromised HRR and increased sensitivity of cancer cells to DNA-damaging agents such as poly (ADP-ribose) polymerase (PARP) inhibitors [20]. Another possible mechanism that can lead to HRD in *ATRIP*-mutated tumors might be the disruption of CHEK1 function, the protein activated by the ATRIP-ATR complex that has a key role in genomic maintenance via the HRR mechanism. Abrogation of CHEK1 function has been shown to inhibit HRR, leading to the accumulation of unrepaired double-stranded breaks and genomic instability [21,22]. It is plausible that compromised localization of the ATR-ATRIP complex on damaged ssDNA may suppress ATR-mediated CHEK1 activation and subsequently lead to HRD. Lastly, ATR inhibitors have been shown to induce replication stress and genomic instability by causing double-stranded breaks at the site of stalled replication forks and inhibiting the repair of the broken DNA. ATR inhibitors render tumor cells sensitive to PARP inhibitors and sensitize BRCA-deficient cancer cells with acquired resistance to PARP inhibitors [21]. Overall, although direct evidence may still be lacking, the existing literature suggests that there could be a connection between loss of ATRIP function and compromised HRR in *ATRIP*-mutated tumor cells.

The critical role of ATRIP in safeguarding progenitor cells against DNA damage has been elucidated through in vivo studies. Matos-Rodrigues et al. demonstrated that conditional inactivation of ATRIP in mouse progenitor cells of the central nervous system and eye led to developmental anomalies such as microcephaly and microphthalmia, culminating in postnatal lethality [23]. Their findings revealed that ATRIP deficiency induces replicative stress and triggers TP53-dependent apoptosis in defective lens progenitor cells. Notably, concurrent inactivation of TP53 in ATRIP-deficient progenitor cells impaired apoptosis, resulting in increased mitotic DNA damage and chromosomal aberrations [23]. These observations underscore ATRIP’s indispensable function in maintaining genomic stability during replication, highlighting its role in preventing DNA damage accumulation and ensuring proper cell cycle progression in progenitor cells.

Future studies should focus on examining tumor phenotypes associated with *ATRIP* mutations in larger carrier cohorts and characterizing the full range of *ATRIP* variant types associated with breast cancer risk. Functional analyses of additional variants, particularly non-founder ones such as the c.1033delC mutation described here, are essential for establishing variant pathogenicity and clarifying genotype–phenotype correlations.

Overall, our report reinforces the emerging concept that *ATRIP* contributes to hereditary breast cancer predisposition and highlights the need for further research to refine risk estimates, strengthen mechanistic understanding, and guide clinical management of individuals harboring *ATRIP* deleterious variants.

## 4. Conclusions

To the best of our knowledge, this is the first report of the detection of a truncating variant in *ATRIP* (NM_130384.3:c.1033delC) among multiple members of an Iranian family with a clustering of breast cancer and negative for mutations in the previously known breast cancer susceptibility genes. *ATRIP* is a newly discovered breast cancer susceptibility gene candidate. Further studies are needed to provide a clearer understanding of the involvement of *ATRIP* in breast cancer susceptibility, enhance the evaluation of risk and management of patients, and potentially discover novel targets for personalized treatment strategies. Meanwhile, any genetic counselling or medical intervention on *ATRIP* mutation carriers based on current knowledge should be performed cautiously.

## Data Availability

The datasets generated during and/or analysed during the current study are not publicly available to protect study participants’ privacy but are available from the corresponding author on reasonable request.
